# Liver X Receptors Enhance Epithelial to Mesenchymal Transition in Metastatic Prostate Cancer Cells

**DOI:** 10.3390/cancers16162776

**Published:** 2024-08-06

**Authors:** Erwan Bouchareb, Sarah Dallel, Angélique De Haze, Christelle Damon-Soubeyrand, Yoan Renaud, Elissa Baabdaty, Marine Vialat, Julien Fabre, Pierre Pouchin, Cyrille De Joussineau, Françoise Degoul, Swapnil Sanmukh, Juliette Gendronneau, Phelipe Sanchez, Céline Gonthier-Gueret, Amalia Trousson, Laurent Morel, Jean Marc Lobaccaro, Ayhan Kocer, Silvère Baron

**Affiliations:** 1 iGReD, CNRS UMR 6293, INSERM U1103, Université Clermont Auvergne, 28, Place Henri Dunant, BP38, 63001 Clermont-Ferrand, France; bou.erwan.pro@outlook.fr (E.B.); sdallel@chu-clermontferrand.fr (S.D.); christelle.soubeyrand-damon@uca.fr (C.D.-S.); yoan.renaud@uca.fr (Y.R.); elissa.baabdaty@uca.fr (E.B.); marine.vialat@uca.fr (M.V.); julienfabre615@gmail.com (J.F.); pierre.pouchin@uca.fr (P.P.); cyrille.de_joussineau@uca.fr (C.D.J.); francoise.degoul@uca.fr (F.D.); swapnil_ganesh.sanmukh@uca.fr (S.S.); juliette.gendronneau@uca.fr (J.G.); phelipe.sanchez@uca.fr (P.S.); celine.gonthier@uca.fr (C.G.-G.); amalia.trousson@uca.fr (A.T.); laurent.morel@uca.fr (L.M.); j-marc.lobaccaro@uca.fr (J.M.L.); 2 Groupe Cancer Clermont Auvergne, 28, Place Henri Dunant, BP38, 63001 Clermont-Ferrand, France; 3 Service d’Endocrinologie, Diabétologie et Maladies Métaboliques, CHU Clermont Ferrand, Hôpital Gabriel Montpied, 63003 Clermont-Ferrand, France

**Keywords:** prostate cancer, epithelial–mesenchymal transition, metastasis, liver X receptors

## Abstract

**Simple Summary:**

As many cancers, prostate cancer is lethal when reaching the metastatic state. Starting from prostate to invade secondary sites, tumour cells undergo structural modifications called epithelial-mesenchymal transition. Molecular mechanisms controlling this phenomenon were studied in vitro and in vivo after modification of LXRs transcription factors activity, which regulate cholesterol metabolism. These studies showed that in a metastatic cell line derived from bone, increased activity of LXR stimulated the EMT processes in vitro and in vivo. We then characterized the associated deregulated genes and identified Amphiregulin as a new target regulated by LXR. The relationship between amphiregulin and EMT was here first reported in prostate cancer and as this protein can be secreted, will be further considered as a possible blood maker for monitoring metastatic occurrence.

**Abstract:**

Prostate cancer (PCa) is one of the most common cancers in men. Metastasis is the leading cause of death in prostate cancer patients. One of the crucial processes involved in metastatic spread is the “epithelial–mesenchymal transition” (EMT), which allows cells to acquire the ability to invade distant organs. Liver X Receptors (LXRs) are nuclear receptors that have been demonstrated to regulate EMT in various cancers, including hepatic cancer. Our study reveals that the LXR pathway can control pro-invasive cell capacities through EMT in prostate cancer, employing ex vivo and in vivo approaches. We characterized the EMT status of the commonly used LNCaP, DU145, and PC3 prostate cancer cell lines through molecular and immunohistochemistry experiments. The impact of LXR activation on EMT function was also assessed by analyzing the migration and invasion of these cell lines in the absence or presence of an LXR agonist. Using in vivo experiments involving NSG-immunodeficient mice xenografted with PC3-GFP cells, we were able to study metastatic spread and the effect of LXRs on this process. LXR activation led to an increase in the accumulation of Vimentin and Amphiregulin in PC3. Furthermore, the migration of PC3 cells significantly increased in the presence of the LXR agonist, correlating with an upregulation of EMT. Interestingly, LXR activation significantly increased metastatic spread in an NSG mouse model. Overall, this work identifies a promoting effect of LXRs on EMT in the PC3 model of advanced prostate cancer.

## 1. Introduction

Prostate cancer (PCa) stands out as one of the most prevalent cancers and is the second-leading cause of cancer-related mortality among men worldwide [1]. The metastatic potential of PCa is directly correlated with its mortality rate. While often manageable when localized, prostate cancer becomes lethal when it spreads beyond the prostate gland. Epithelial–mesenchymal transition (EMT) is a fundamental process associated with the metastatic progression of cancer [2,3,4,5,6]. During EMT, cancerous cells acquire high migration and invasion potential, enabling them to detach from the primary tumor and form metastases in distant organs [2], such as lymph nodes, bones, and lungs in PCa. Liver X Receptors (LXRs) belong to the nuclear receptor superfamily and are involved in various physiological functions. They are encoded by two distinct genes, *NR1H3* and *NR1H2*, encoding LXRα and LXRβ, respectively. Mainly known for their role in controlling cholesterol metabolism, facilitating cholesterol efflux from cells [7], and lipogenesis [8], LXRs also play a significant role in regulating steroidogenesis, glucose metabolism, and immune responses [9,10,11]. LXRs are emerging as important players in the control of EMT, whether in physiological processes associated with development and morphogenesis or in pathologies such as cancer. Indeed, LXR agonists have been shown to suppress pulmonary fibrosis [12] and block myofibroblast differentiation [13]. Some EMT-associated transcription factors, such as SNAIL, TWIST1, and zing finger E-box binding homeobox 1/2 (ZEB1/2), can be downregulated following LXR activation [14,15], and GW3965 treatment, a synthetic LXR agonist, reverses gefitinib resistance, an inhibitor of the epidermal growth factor receptor (EGFR), in non-small cell lung cancer by inhibiting Vimentin [16]. In vivo studies have shown an upregulation of SNAIL in mouse prostates knocked down for both LXRs [15]. Altogether, these observations highlight the inhibitory action of LXRs on EMT. However, the role played by LXRs in the control of EMT in PCa still remains poorly understood. Here, we demonstrate that the activation of LXRs increases the migration and invasion capacities of PC3 prostate cancer cells. This phenotype is associated with an accumulation of specific EMT markers, Vimentin and Amphiregulin. Furthermore, in vivo experiments conducted on immunocompromised mice showed that LXR activation exacerbated the aggressive phenotype of PC3 cells, leading to an increase in the number of metastases. This work suggests that LXRs could control tumor progression in advanced PCa by promoting EMT.

## 2. Methods

### 2.1. Culture and Treatment

Human prostate cancer cell lines (PC3 (ATCC CRL-1435), DU145 (ATCC HTB-81), and LNCaP (ATCC CRL-1740)) were obtained from American Type Culture Collection (ATCC, LGC Standards, Molsheim, France). Cells were maintained in RPMI-1640 supplemented with 10% FBS, penicillin/streptomycin (100 µg/mL) and L-Glutamine (2 mM) and were kept in a humidified incubator at 37 °C with 5% CO_2_. Cells were treated with DMSO (Sigma-Aldrich, L’Isle d’Abeau, France) as the control, with LXR ligand GW3965 (Bertin Bioreagent, Montigny le Bretonneux, France), T0901317 (Bertin Bioreagent), or RXR ligand 9*cis* retinoic acid (Bertin Bioreagent). The siRNAs targeting AREG were as follows: AREG, 5′-UAUUUCCUGACGUAUUGUCUU-3′. The siRNA control sequences were 5′-UGCGCUAGGCCUCGGUUGC-3′. All siRNAs were purchased from Eurogentec, Liege, Belgium. A total of 30 pmol of each siRNA was transfected into PC3 cells using Lipofectamine RNAiMAX Reagent according to the manufacturer’s procedure (Invitrogen, Thermo Fisher Scientific, Inc., Waltham, MA, USA).

### 2.2. Western Blot Analysis

Proteins were extracted using lysis buffer containing HEPES 20 mM, NaCl 0.42 M, MgCl_2_ 1.5 mM, EDTA 0.2 mM, and NP40 1% supplemented with protease inhibitors, PMSF 1 mM (Sigma-Aldrich), Complete 1× (Roche Diagnostics, Meylan, France), and phosphastase inhibitor cocktail, NaF 1 mM (Sigma-Aldrich), Na_3_VO_4_ 1 mM (Sigma-Aldrich), and 0.5 mM DTT (Sigma-Aldrich). Lysates were centrifuged at 4 °C for 15 min at 15,000× *g*. The protein content was determine using the Bradford reagent (Bio-Rad, Marnes-la-Coquette, France). Protein extracts were denatured using Laemmli buffer (Bio-Rad), heated for 5 min at 95 °C, and resolved by SDS-PAGE. Proteins were next transferred onto nitrocellulose Hybond-ECL membrane (GE Healthcare Life Sciences, Velizy-Villacoublay, France). Membranes were blocked using non-fat dry milk diluted in TBS-Tween 0.1% (TBS-T) buffer for 1 h and then incubated with primary antibodies, according to the list available in Table 1, overnight at 4 °C in TBS-T containing 5% BSA or non-fat dry milk. Next, the membranes were washed using TBS-T and incubated with anti-rabbit or anti-mouse (Abliance, Compiègne, France) secondary antibody conjugated to horseradish peroxidase for 1 h. β-ACTIN was used as the loading control. The revelation was performed by using Clarity Western ECL substrate (Bio-Rad) and captured with the ChemiDoc MP Imaging System (Bio-Rad).

### 2.3. RT-qPCR

RNAs were extracted from PCa cells lines using the Direct-zol kit (Ozyme, Saint Cyr l’Ecole, France), following the manufacturer’s instructions. RNAs were converted into cDNA by retro-transcription using the MMLV enzyme and random primers (Promega, Charbonnières-les-Bains, France). Real-time PCR amplifications were performed with SYBR qPCR Premix Ex Taq II Tli RNase H+ Bulk (Takara, Saint-Germain-en-Laye, France) to measure the levels of specific genes. For each experiment and primer pair, the efficiency of PCR reactions was evaluated by the amplification of serial dilutions of a mix of cDNAs by using the specific primer pairs available in Table 2. Relative gene expression was normalized to *ACTIN* gene expression by the ΔΔCt method.

### 2.4. Migration and Invasion Assay

PC3 cells were seeded in sterile transparent 96-well Incucyte Imagelock plates at 17,000 cells/well in 100 µL of complete culture medium and incubated overnight at 37 °C and 5% CO_2_. The next day, a scratch wound was performed in the confluent cell monolayer of each well using WoundMaker (Essen Bioscience, Newark Close, Royston Hertfordshire, UK) following the manufacturer’s protocol. Briefly, cells were treated with low-serum media (1% FBS) containing DMSO or GW3965, and the plates were placed in an Incucyte device (Sartorius, Essen Bioscience) and incubated at 37 °C and 5% CO_2_. The images of cell migration were taken using an inverted light microscope at 20× magnification at different time points after the injury (0–48 h, every 2 h). The experiments were conducted in triplicate for each condition. The images were analyzed quantitatively using the Incucyte ZOOM™ live cell imaging system (Sartorius, Essen Bioscience) that automatically calculates using a mathematical model to calculate the percentage of wound closure.

Invasion assays were performed with Transwell cell culture inserts (Transparent PET membrane, 24 wells, pore size: 8 µm, Corning, Bagneaux sur Loing, France). Prior to cell seeding, the inserts were coated with Matrigel matrix basement membrane (Corning, REF 356237). A total of 5 × 10^5^ cells were suspended in the upper chamber of the Transwell inserts. The upper chamber was filled with serum-free RPMI-1640 medium, supplemented with either DMSO or GW3965. For chemoattraction, the lower chamber was filled with RPMI-1640 medium containing 10% FBS, along with DMSO or GW3965. After incubating for 24 h, the cells were washed twice with PBS. Subsequently, a cotton swab soaked in PBS was used to remove the cells from the upper chambers. Invading cells were fixed with 100% methanol and stained using a 0.1% crystal violet solution (Sigma-Aldrich). To assess the invasion, photographs of all the inserts were captured using a light microscope (Leica MZ75, Nanterre, France). The invasion index was determined by calculating the total covered area in the insert using Image J software V2.14.0/1.54f [17].

### 2.5. Histological and Immunofluorescence Analysis

Paraffin-embedded tissue sections were sectioned for hematoxylin and eosin staining. Alternatively, immunohistochemistry was performed on paraffin-embedded tissues after antigen retrieval, if necessary, by boiling for 20 min in sodium citrate 10 mM, Tween 0.05% (pH 6); Tris 10 mM, EDTA 1 mM (pH 9.0); or Vector Unmasking Solution (H3300, Vector Laboratories, Burlingame, CA, USA), depending on the primary antibody. The histological sections were then blocked in 1% BSA for 1 h at room temperature. Next, slides were incubated at 4 °C overnight with primary antibodies at the indicated concentrations (Table 1). Primary antibodies were detected with appropriate polymers (ImmPress Polymer Detection Kit, Vector Laboratories). Polymer-coupled HRP activity was then detected with either Vectastain ABC (PKD4000, Vector Laboratories) for brightfield images or Tyramide SuperBoost Kits with Alexa Fluor Tyramide for fluorescence (Invitrogen, Waltham, MA, USA). Nuclei were counterstained with hematoxylin or Hoechst (Invitrogen). Images were acquired with a Zeiss AxioImager with Apotome2 and a Zeiss Axioscan Z1 slide scanner (Zeiss, Oberkochen, Germany). Cell circularity was determined with the MorphoLibJ plug-in using Image J software [17].

### 2.6. Animals and Tumor Xenografts

All experiments were approved by the Institutional Animal Care and Use Committee (CEMEAA). NOD-Scid-γ mice were provided by Charles River, and the *LXRαβ*-/- mouse analyses were conducted using samples collected in a previous study following 3R recommendations [18]. Animals were housed in a controlled environment with a 12 h light and 12 h dark cycle. The mice were provided with a standard global diet (2016S, Harlan, Gannat, France) and water ad libitum. Xenografts were performed using 12-to-15-week-old male NOD-Scid-γ mice using surgical implantation into the anterior lobe of the prostate. From the various human prostate cancer cell lines (PC3, DU145, and LNCaP), 1 × 10^5^ cells were resuspended in PBS1X and encapsulated in MATRIGEL (Corning, REF 356237) *vol*/*vol* for a final volume of 10 µL prior to implantation into the anterior lobe of the prostate. One month later, xenografted tissues were collected after necropsy and fixed in Paraformaldehyde 4% and embedded in paraffin. Paraffin sections were prepared for immunohistochemical studies. Regarding GW3965 mouse treatment, following the implantation of PC3-GFP cells into the anterior lobe of the prostate, mice were randomized into 2 groups and treated three times a week for 1 month with *i.p.* injections of GW3965 10 mg/kg (Bertin Bioreagent) or DMSO (Sigma-Aldrich) as the vehicle.

### 2.7. RNA Sequencing Analysis

Library preparation was performed in the GenomEast platform at the Institute of Genetics and Molecular and Cellular Biology using Illumina Stranded Total RNA Prep Ligation with Ribo-Zero Plus—Reference Guide—PN 1000000124514. Total RNA-Seq libraries were generated from 200 or 500 ng of total RNA using the Illumina Stranded Total RNA Prep, Ligation with Ribo-Zero Plus kit and IDT for Illumina RNA UD Indexes, Ligation (Illumina, San Diego, CA, USA), according to the manufacturer’s instructions. Briefly, abundant ribosomal RNAs were depleted by hybridization to specific DNA probes and enzymatic digestion. The depleted RNAs were purified and fragmented using divalent cations at 94 °C for 8 min. After random hexamer annealing, fragmented RNAs were then reverse transcribed into first-strand complementary DNA (cDNA). Second-strand cDNA synthesis further generated blunt-ended double-stranded cDNA and incorporated dUTP in place of dTTP to achieve strand specificity by quenching the second strand during amplification. Following A-tailing of DNA fragments and the ligation of pre-index anchors, PCR amplification was used to add indexes and primer sequences and to enrich DNA libraries (30 s at 98 °C; [10 s at 98 °C, 30 s at 60 °C, 30 s at 72 °C] × 12 cycles; 5 min at 72 °C). Surplus PCR primers were further removed by purification using SPRIselect beads (Beckman-Coulter, Villepinte, France), and the final libraries were checked for quality and quantified using capillary electrophoresis. Libraries were sequenced on an Illumina NextSeq 2000 sequencer as single-read 50 base reads. Image analysis and base calling were performed using RTA version 2.7.7 and BCL Convert version 3.8.4. Reads were aligned using mouse reference genome mm10 with Hisat2 v2.2.1. Reads counts were reported for each annotated gene using R V2024.04.1+748 and converted into RPKM. Principal component analyses were generated for each dataset using the plotPCA function from R package Deseq2 V1.42.1. Heatmaps were generated using R « gplots » v3.0.1. and “pheatmap 1.0.12” packages. Gene expression datasets were analyzed using GSEA 4.1.0 with genesets from the MSigDB Hallmark gene set. The dataset is available using GEO accession GSE245578.

### 2.8. Statistical Analysis

Data are expressed as mean ± SD. Statistical analyses were performed with the Mann–Whitney test. Values of *p* < 0.05 were considered significant. Statistical analyses were performed with GraphPad Prism Software v8.0.1.

## 3. Results

### 3.1. Characterization of the Prostatic Tumor Cell Lines LNCaP, DU145, and PC3 Reveals Varying Degrees of EMT Status

**Figure 1:**
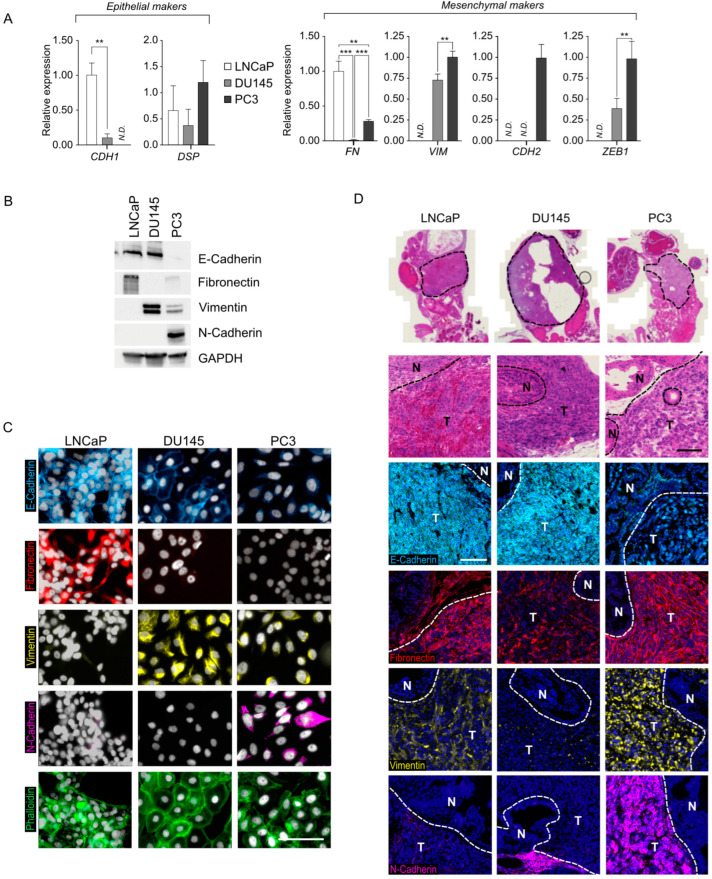
Characterization of epithelial to mesenchymal status of LNCaP, DU145, and PC3 prostatic cancer cell lines. (**A**) Relative expression of epithelial markers *CDH1* and *DSP* and mesenchymal markers *FN*, *VIM*, *CDH2,* and *ZEB1* was analyzed and normalized to *GAPDH* in LNCaP, DU145, and PC3 cells (n = 6 per condition). Data are expressed as the means ± SD. Mann–Whitney test statistical analysis; ** *p* < 0.01, *** *p* < 0.001. (**B**) Representative Western blots of E-Cadherin, Fibronectin, Vimentin, and N-Cadherin accumulation in LNCaP, DU145, and PC3 cells. GAPDH was used as a loading control. (n = 4 per condition.) (**C**) Immunofluorescence of LNCaP, DU145, and PC3 cells stained for E-cadherin (*blue*), Fibronectin (*red*), Vimentin (*yellow*), and N-Cadherin (*purple*). Actin was stained using Phalloidin (*green*). Nuclei were stained using Hoescht (*white*). Scale bar: 100 µm. (**D**) HE-stained sections and immunofluorescence detection of E-cadherin (*blue*), Fibronectin (*red*), Vimentin (*yellow*) and N-Cadherin (*purple*) in primary tumor site following prostatic orthotopic xenograft with LNCaP, DU145, and PC3 cells. Tissues were analyzed 1 month following implantation. N: Normal. T: Tumor. Scale bar: 200 µm. Original western blots are presented in File S3.

To investigate the role of LXRs in the epithelial–mesenchymal transition in prostate tumor cells, we chose three widely used prostatic cell models: the LNCaP, DU145, and PC3 cell lines. We conducted a comprehensive characterization of the epithelial–mesenchymal profile for each of them. Epithelial or mesenchymal status is not a binary criterion but rather a *continuum* defining different stages of partial EMT. Initially, we assessed the expression levels of epithelial status-specific marker genes, *CDH1* encoding E-Cadherin and *DSP* encoding Desmoplakin (Figure 1A), confirming previous observations that placed LNCaP cells with the strongest epithelial identity and PC3s with the weakest [19]. We then examined the expression of genes associated with the mesenchymal phenotype, such as *FN*, *VIM*, *CDH2*, and *ZEB1* encoding Fibronectin, Vimentin, N-Cadherin proteins and Zing Finger E-Box Binding Homeobox 1 (ZEB1) transcription factor, respectively. (Figure 1A). Except for the expression profile of the *FN* gene, our results aligned with previous data [19,20,21,22], indicating a more advanced profile toward the mesenchymal phenotype for PC3 and, to a lesser extent, DU145, with LNCaP displaying a very weak mesenchymal phenotype. These gene expression findings were further validated by protein accumulation analysis (Figure 1B), showing the accumulation of E-Cadherin, Fibronectin, Vimentin, and N-Cadherin proteins correlating with the respective gene expression profiles for each cell line. The subcellular localization and accumulation of these proteins within cells are critical features regarding epithelial and/or mesenchymal identity. Immunofluorescence experiments confirmed the expected localization and accumulation of each marker, consistent with the RT-qPCR expression data and protein accumulation observed in Western blot analyses (Figure 1C). Subsequently, we explored epithelial–mesenchymal identity in vivo using an orthotopic graft model on NSG-mice to assess whether the EMT phenotype was stable and cell-autonomous or dependent on the cellular environment (Figure 1D and Figure S1). LNCaP and DU145 cells accentuated their epithelial identity, as evidenced by a significant accumulation of membrane E-cadherin and a weak or null accumulation of Vimentin and N-Cadherin. In contrast, PC3 cells exhibited a strong mesenchymal phenotype with a low accumulation of E-Cadherin at the nuclear level and a high accumulation of Vimentin and N-Cadherin. Overall, we observed a gradation of the LNCaP, DU145, and PC3 cell lines from an epithelial to mesenchymal status [19,20,21,22], with a pronounced dichotomy in the context of orthotopic grafts, where LNCaP and DU145 had the closest epithelial identity, and the PC3 line mostly displayed a mesenchymal identity.

### 3.2. LXR Signaling Is Functional in Prostate Tumor Cell Lines

**Figure 2:**
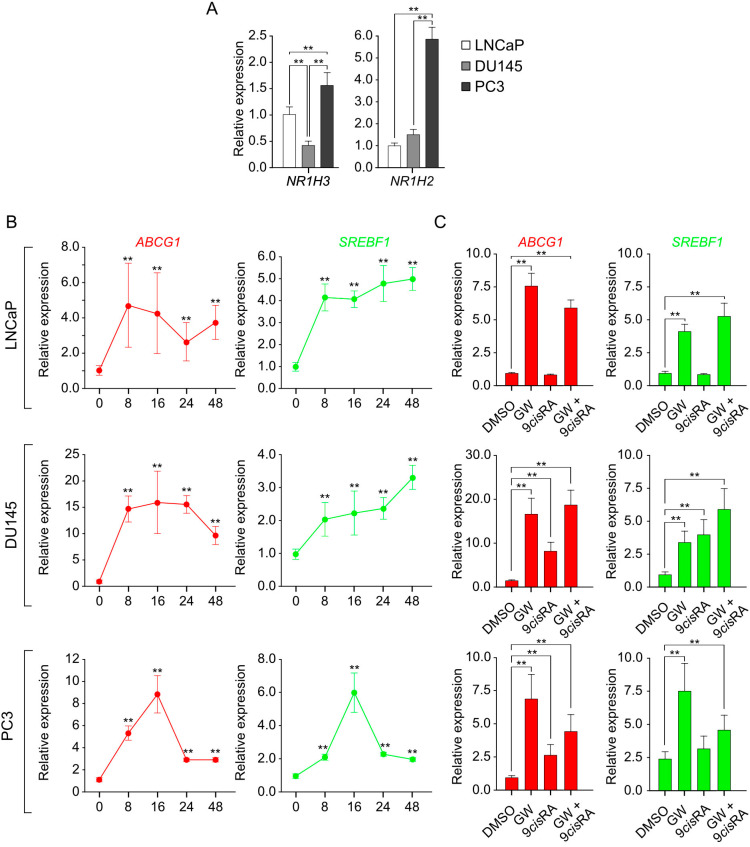
LXR signaling is functional in each prostatic cancer cell line. (**A**) *NR1H3* and *NR1H2* expression levels were normalized to *GAPDH* in LNCaP, DU145, and PC3 cells (n = 6 per condition). (**B**) *ABCA1* and *SREBF1* kinetic expression levels were normalized to *GAPDH* in LNCaP, DU145, and PC3 cells treated with LXR agonist GW3965 for 8 h, 16 h, 24 h, and 48 h (n = 6 per condition). (**C**) *ABCA1* and *SREBF1* expression levels were normalized to *GAPDH* in LNCaP, DU145, and PC3 cells treated with LXR agonist GW3965, RXR agonist 9*cis*RA (9cis retinoic acid), and a combination of both for 16 h (n = 6 per condition). Data are expressed as the means ± SD. Mann–Whitney test statistical analysis; ** *p* < 0.01.

Subsequently, we sought to understand the extent to which LXR signaling could be activated in each lineage. Using RT-qPCR, we analyzed the expression of the *NR1H3* and *NR1H2* genes encoding LXRalpha and LXRbeta, respectively (Figure 2A). Overall, each cell line expresses both receptors. *NR1H3* is mostly expressed in PC3 compared to LNCaP, with a lower rate for DU145 compared to the other two cell lines. PC3 cells express more *NR1H2* than the other two cell lines. We then explored the functional activity of LXRs in each cell line by monitoring the expression of two endogenous canonical target genes of LXR/RXR heterodimers by RT-qPCR, the *ABCG1* and *SREBF1* genes encoding the cholesterol transporter ATP Binding Cassette Subfamily G Member 1 (ABCG1) [23] and the transcription factor Sterol Regulatory Element-Binding Transcription Factor 1 (SREBP1c) [24] (Figure 2B,C). These proteins are, respectively, involved in cholesterol efflux and fatty acid synthesis. To assess this, we conducted treatment kinetics with the synthetic LXR ligand GW3965 over 48 h (Figure 2B) and the sequential activation of LXR, RXR, or the entire heterodimer by treating cells for 24 h with GW3965 alone, 9*cis* retinoic acid alone (RXR ligand), or a combination of both (Figure 2C). The two genes showed induced expression in response to LXR agonist treatment in all three tested cell lines. Regarding the expression profiles of the *ABCG1* gene, we observed a greater relative induction in DU145 and PC3 cells compared to LNCaP cells, which was not found for *SREBF1* (Figure 2B). Furthermore, we noted a time-dependent induction with “plateau” profiles for LNCaP and DU145 cells for the two analyzed genes, while their expression was transient in PC3 cells (Figure 2B). The sequential inductions with GW3965 or 9*cis* retinoic acid showed effective stimulation by both types of ligands in DU145 and PC3 cells for *ABCG1* expression and in DU145 for *SREBF1* expression, indicating that both receptors of the heterodimer are functional, but without additive or synergistic effects (Figure 2C). Surprisingly, we did not observe any effect of 9*cis* retinoic acid alone or in combination with GW3965 in LNCaP cells whatever the LXR target gene monitored (Figure 2C). All these results indicate that LXR signaling effectors are present and active in all three cell lines studied.

**Figure 3:**
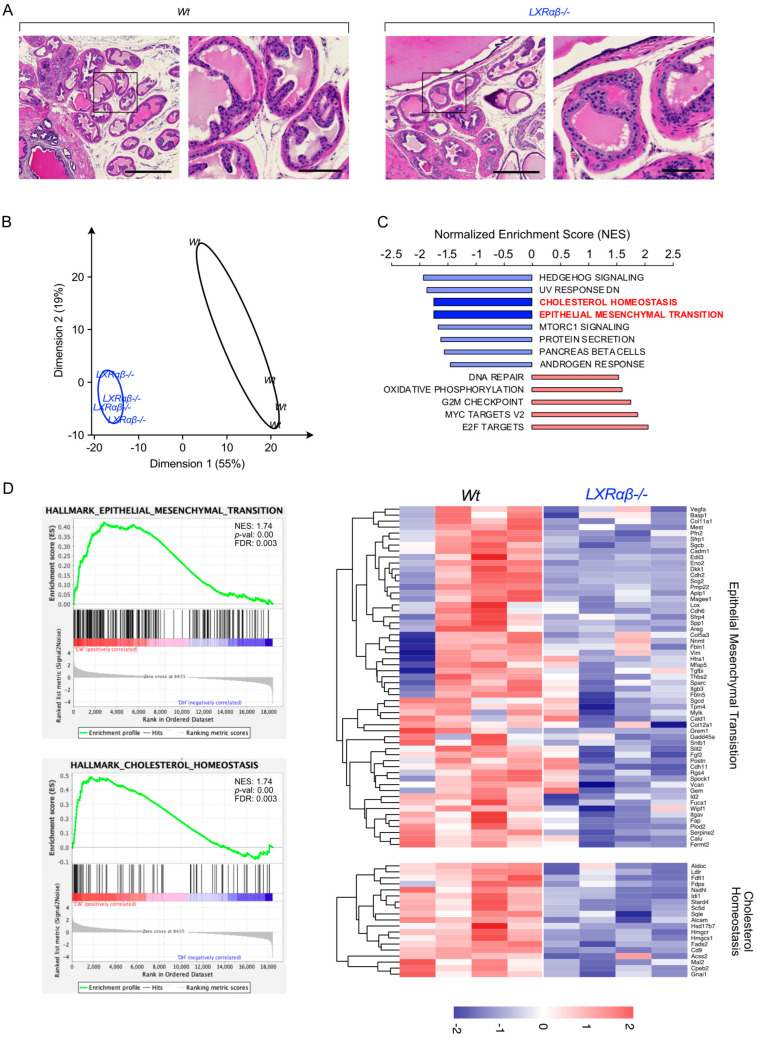
LXR-deficient mouse prostate reveals a decrease in mesenchymal transcriptomic signature. (**A**) HE-stained sections of dorsal prostates from WT mice and *Lxr*αβ-/- mice. Scale bars: upper panel: 500 µm; lower panel: 100 µm (**B**) Principal component analysis from RNAseq datasets of prostate WT mice and LXRαβ-/- mice capturing 55% (dimension 1) and 19% (dimension 2) of the data variability. (**C**) GSEA hallmark enrichment scores were ranked comparing signatures from WT and *Lxr*αβ-/- mouse prostates. (**D**) GSEA analysis plots for epithelial–mesenchymal transition and cholesterol homeostasis gene clusters and corresponding leading-edge gene set heatmap for each of them (NES: normalized enrichment score, FDR: false discovery rate).

LXR-null mouse prostates reveal a deficiency of a mesenchymal signature. To address the role of LXRs in the EMT process in prostate cells, we chose to investigate the phenotype of the prostatic lobes of wild-type mice versus DKO LXR (mice invalidated both for *Nr1h2* and *Nr1h3* encoding LXRα and LXRβ, respectively). Indeed, the EMT process plays a critical role during prostate ontogenesis. Histological analysis of the prostate of DKO LXR mice does not reveal any major defect in the establishment of the various cellular compartments, particularly in the epithelial and the stromal ones (Figure 3A). To further explore a potential effect of the absence of LXRs on the prostate phenotype, we analyzed the transcriptomic data available. PCA analysis displays that prostates from DKO LXR mice exhibit a specific transcriptomic signature (Figure 3B). GSEA hallmark analysis reveals, as expected, a deregulation of genes involved in cholesterol homeostasis. The deregulation of cholesterol-associated genes confirms the intrinsic effect of LXR ablation in this tissue. In parallel, DKO LXR prostatic tissue reveals a strong deregulation of a gene cluster linked to EMT (Figure 3C,D). Indeed, a careful analysis of the gene list composing the leading edge of this EMT cluster reveals a strong decrease in their expression. This finding links, from a molecular point of view, the absence of LXRs to the deregulation of the genes involved in EMT. In conclusion, although the prostatic tissue of DKO LXR mice presents a normal phenotype compared to that of wild types, the latter is characterized by a coordinated reduction in genes involved in the EMT process.

### 3.3. Migration and Invasiveness Capacities Are Enhanced by LXR Activation and Correlated with Vimentin Accumulation

**Figure 4:**
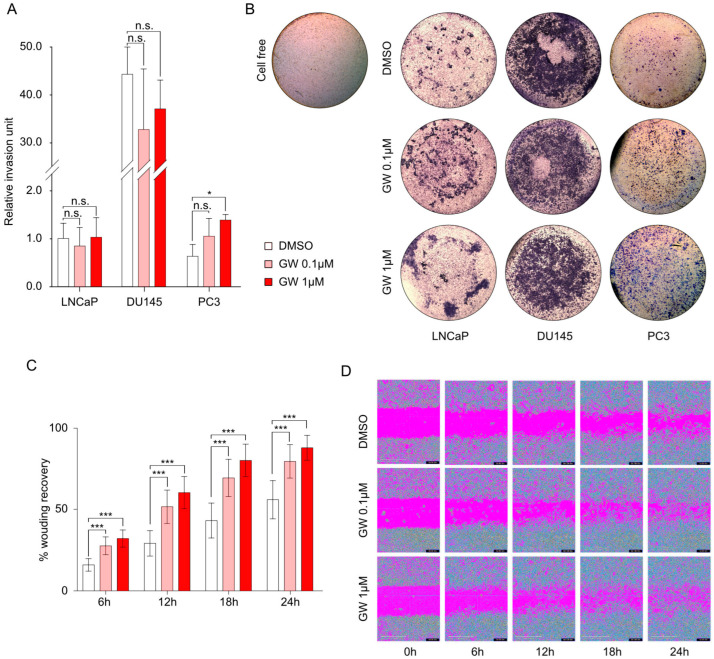
LXR activation by GW3965 enhances the migration and invasion properties of PC3 cells. (**A**) Invasion assays were performed using Boyden chambers. PC3, DU145, and LNCaP cells seeded in culture medium without FBS were placed in the upper chamber previously coated with Matrigel. Medium with FBS was added in the lower chamber and the cells were treated with GW3965 (0.1 or 1 µM) for 24 h for each cell line. (**B**) Representative pictures of invaded cells. (**C**) Wound healing assays were performed to determine the effect of LXR activation on migration capacities. PC3 cells were treated with GW3965 (0.1 or 1 µM) and analyzed following 6 h, 12 h, 18 h, and 24 h of treatment. For each time point, relative wound closure was evaluated using Incucyte station. (**D**) Representative pictures of migrated cells. Scale bars: 400 µm. Data are expressed as the means ± SD. Mann–Whitney test statistical analysis; * *p* < 0.05, *** *p* < 0.001.

**Figure 5:**
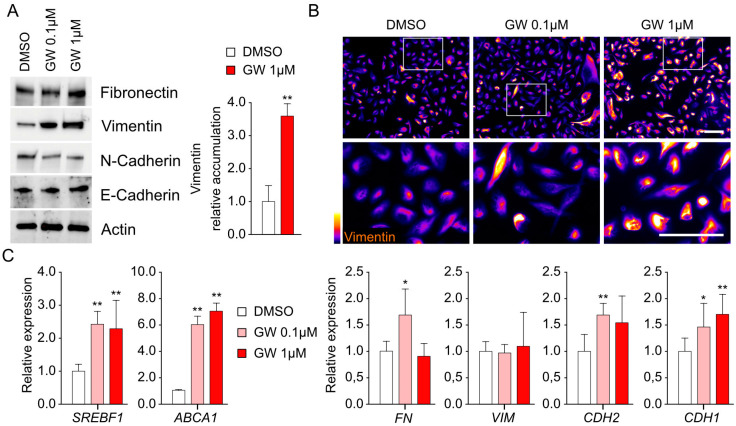
GW3965 treatment stimulates Vimentin accumulation in PC3 cells. (**A**) Representative Western blots of Fibronectin, Vimentin, N-Cadherin, and E-Cadherin accumulation using PC3 cells treated with DMSO (control) and GW3965 (0.1 or 1 µM). VIM accumulation is significantly increased in PC3 cells treated with 1 µM of GW3965. GAPDH was used as a loading control. (**B**) Immunofluorescence of PC3 cells stained for VIM (*Fire scale*). Scale bar: 100 µM. (**C**) *ABCA1, SREBF1* (LXR target genes), and *FN*, *VIM*, *CDH1*, and *CDH2* (mesenchymal genes) expressions were normalized using *GAPDH* in cells treated with DMSO or GW3965 (0.1 and 1 µM). (n = 6 per condition). Data are expressed as the means ± SD. Mann–Whitney test statistical analysis; * *p* < 0.05, ** *p* < 0.01. Original western blots are presented in File S3.

Given the observations collected in the mouse prostate highlighting the link between LXR and EMT, we questioned whether LXR activation could modify the invasive and migratory properties of cells from the LNCaP, DU145, and PC3 human cell lines. To investigate this, we conducted invasion tests using a Boyden chamber (Figure 4A,B). We observed that treatment with increasing doses of GW3965 had little effect on LNCaP and DU145 cells. The three cell lines harbored different intrinsic capacities to invade under basal conditions (Figure 4A,B). However, only PC3 cells were revealed to be sensitive to GW3965 treatment, with a significant increase in the invasive properties when induced by 1 µM of GW3965. Subsequently, we aimed to determine if these invasive effects were dependent on the migration activity of PC3 cells. Thus, we conducted scratch assays to evaluate migration in response to LXR activation (Figure 4C,D). Consistent with the data obtained on PC3 cells in Boyden chambers, we observed increased migratory capacities of these cells in the presence of GW3965. To understand whether these observations were linked to a modification of the EMT properties, we analyzed the protein accumulation profiles of mesenchymal markers (Fibronectin, Vimentin, and N-Cadherin) compared to E-Cadherin accumulation (Figure 5A). We were able to demonstrate a significantly increased accumulation of Vimentin in response to GW3965 treatment (Figure 5A). These observations were confirmed by the immunolocalization of Vimentin on PC3 cells (Figure 5B). Unexpectedly, an analysis of the expression of genes encoding these same markers (*FN*, *VIM*, and *CDH2*) showed only very slight variations in expression (Figure 5C), while known target genes *SREBF1* and *ABCG1* exhibited expected expression stimulation (Figure 5C). This is particularly evident in the case of the *VIM* gene, encoding Vimentin, which remained unchanged regardless of the treatment. This result suggests an indirect post-transcriptional control of Vimentin accumulation by LXR activation without any effect regarding Vimentin promoter regulation. Overall, the results show that in PC3 cells, which have the most advanced status toward mesenchymal identity, the pharmacological activation of LXR leads to an increase in invasion and migration properties associated with the accumulation of Vimentin.

### 3.4. Transcriptomic Signature Associated with LXR Stimulation Reveals Amphiregulin-Encoded Gene as a Potential Target

**Figure 6:**
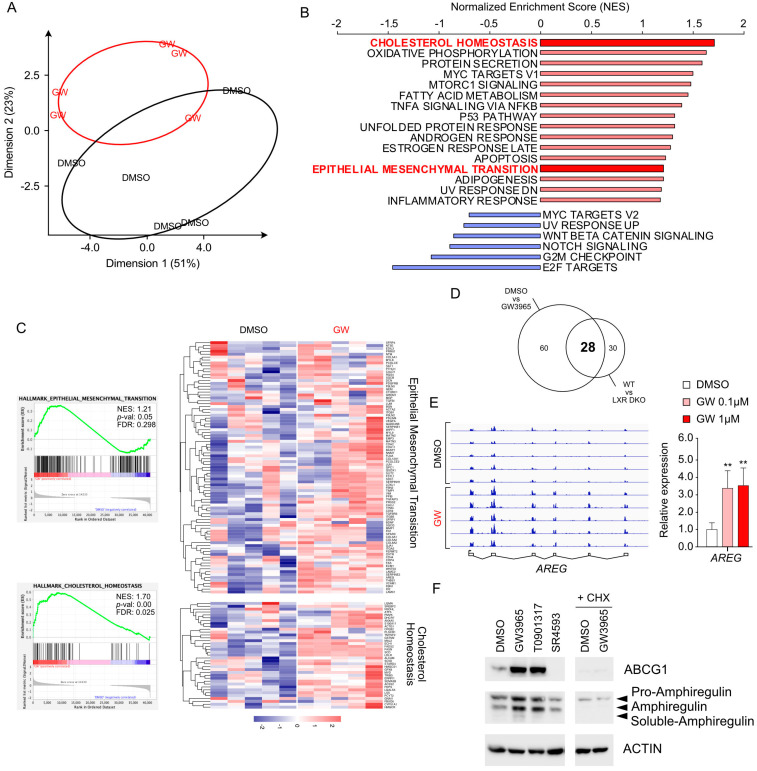
Molecular signature of GW3965 treatment reveals *AREG* as a potential target gene to control epithelial to mesenchymal transition. (**A**) Principal component analysis of PC3 cells treated with DMSO vs. GW3965 (1 µM) for 24 h, capturing 51% (dimension 1) and 23% (dimension 2) of the data variability. (**B**) GSEA hallmark rankings of enrichment scores in PC3 cells treated or not with GW3965 were plotted. (**C**) GSEA analysis plots for epithelial–mesenchymal transition and cholesterol homeostasis gene clusters and a corresponding leading-edge gene set heatmap for each of them. (**D**) Venn diagram summarizing the overlap between DRGs of PC3 cells treated with DMSO vs. GW3965 (left circle) and DRGs of prostates from WT vs. *Lxr*αβ-/- mice (right circle). This analysis leads to the identification of 28 genes deregulated in both datasets. (**E**) RNA-seq enrichment profiles for *AREG* locus coding sequences in PC3 cells treated with DMSO or with GW3965 (0.1 or 1 µM) for 24 h (n = 5 per condition) correlated with the RT-qPCR analysis of *AREG* expression using a similar experimental design (n = 6 per condition) (**F**) Representative Western blots of Amphiregulin accumulation from three independent experiments in PC3 cells treated with DMSO, GW3965 (1 µM), T0901317 (1 µM), or SR4593 (1 µM). Alternatively, PC3 cells treated with DMSO or GW3965 (1 µM) with cycloheximide (CHX) (50 µg/mL), an inhibitor of translation (n = 3 per condition). ABCG1 protein accumulation was monitored as a control of LXR activation, with ACTIN as a loading control. Three forms of Amphiregulin (AREG) were detected: Pro-Amphiregulin, Mature Amphiregulin, and the soluble form (Black arrows). Data are expressed as the means ± SD. Mann–Whitney test statistical analysis; ** *p* < 0.001. Original western blots are presented in File S3.

To identify the molecular mechanisms downstream of LXRs that may control the EMT process, we chose to conduct a transcriptomic analysis using PC3 cells. Data analysis by PCA shows a clear distinction between the replicates treated with GW3965 and the control replicates treated with DMSO (Figure 6A). GSEA hallmarks reveal, as expected, the emergence of the cholesterol homeostasis gene cluster (Figure 6B,C). Additionally, we observe the presence of an EMT cluster significantly associated with GW3965 exposure (Figure 6B,C). The leading-edge projection of this cluster in the heatmap confirms an upregulation of expression induced by GW3965. To isolate genes sensitive to pharmacological induction as well as dependent on LXR, we decided to intersect the EMT cluster obtained in the DKO LXR model and PC3 cells induced by GW3965. A Venn diagram analysis reveals a list of 28 genes overlapping GW3965 sensitivity and the LXR DKO’s clusters (Figure 6D, Table 3). Among these 28 genes, the *AREG* gene (ENSG00000109321) encoding Amphiregulin particularly drew our attention. Amphiregulin is related to epidermal growth factor (EGF) and transforming growth factor (TGF) and interacts with EGF and TGF-alpha receptors to control epithelial cell growth [25]. Amphiregulin plays crucial role in tissue repair, inflammation, and immunity [26]. Amphiregulin is also involved in the control of EMT [27], especially by regulating the half-life of Vimentin [28]. We confirmed the upregulation of AREG expression through bedgraph analysis and RT-qPCR (Figure 6E). Furthermore, we also observed an increase in the accumulation of Amphiregulin by Western blot in response to GW3965 and T0901317 (synthetic LXR ligand) (Figure 6F). In agreement with a regulation by LXR, the inverse agonist SR4593 led to a significant decrease in Amphiregulin accumulation. Treatment with cycloheximide (CHX), a translation inhibitor, confirmed that AREG is a direct target of LXR. Consistent with this, ABCG1 protein accumulation, as a control of LXR activity status, fully mirrored the Amphiregulin accumulation profile (Figure 6F). In conclusion, the comprehensive analysis of the transcriptome of PC3 cells induced by GW3965 led to the identification of AREG as a potential link between LXR activation and EMT.

### 3.5. Pharmacological Targeting of LXR Results in Increased Metastatic Properties of PC3 Cells In Vivo

**Figure 7:**
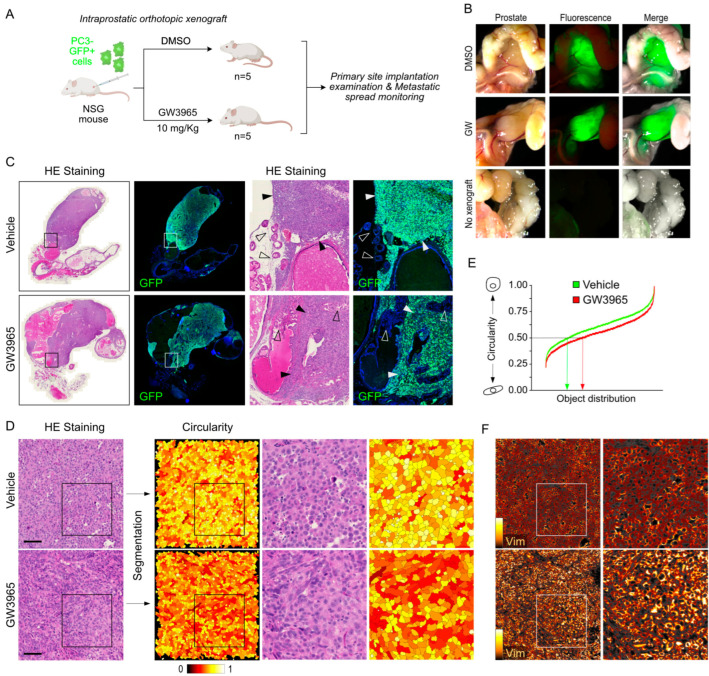
GW3965 increases Vimentin accumulation and the spindle-shape PC3 cell phenotype in a model of NSG orthotopically grafted mice. (**A**) Experimental procedure to study the metastatic spread of human advanced prostate cancer cells in NSG host mice. PC3-GFP were xenografted in NSG mice. One week after the surgery, mice were treated (intraperitoneal injection) with either DMSO or an LXR agonist (GW3965, 10 mg/kg), twice a week. Mice were sacrificed 5 weeks post-implantation, and prostates, lungs, lymph nodes, and kidneys were collected. (**B**) Representative pictures of implantation primary site (prostate) obtained during necropsy. GFP-fluorescence and HE-stained sections of the prostate confirm the graft success compared to non-grafted mice. (**C**) GFP immunodetection on the primary site of implantation observed using mice treated with DMSO or GW3965. Higher magnification exhibits the tumor border (filled arrows) compared to the normal tissue (open arrows) negative for the GFP. (**D**,**E**) Hematoxylin and eosin staining was used to analyze cellular spindle shape in vehicle- or GW3965-treated mice. Cell circularity was revealed with a red hot scale after segmentation and quantified using image J software. (**F**) Vimentin accumulation was detected (orange hot scale) in tumors from both vehicle- or GW3965-treated mice. Scale bar: 100 µm.

**Figure 8:**
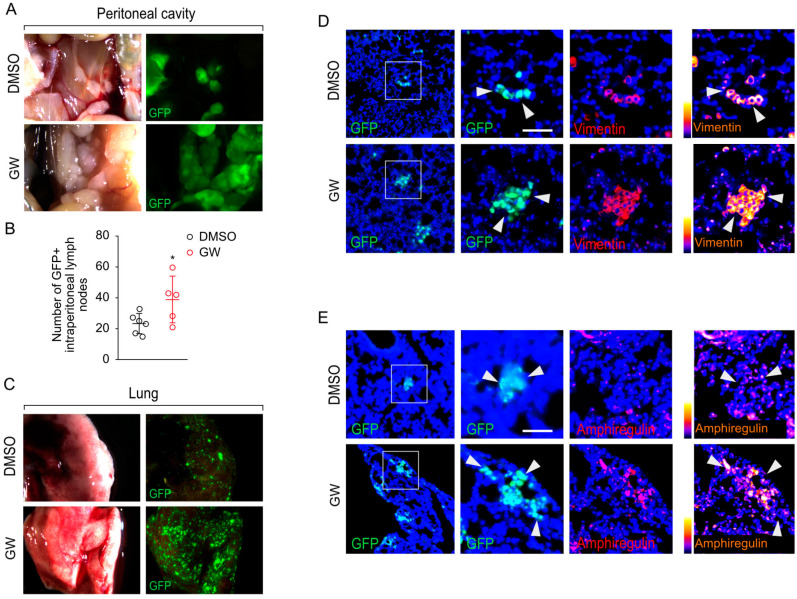
GW3965 increases PC3 invasiveness in NSG orthotopically grafted mice. (**A**) Representative pictures of the peritoneal cavity from vehicle- or GW3965-treated PC3-grafted mice. (**B**) Number of metastatic lymph nodes counted in the peritoneal cavity during necropsy. (**C**) Representative pictures of lungs obtained during necropsy. GFP-fluorescence exhibits an increase in fluorescent signals in mice treated with GW3965. (**D**,**E**) Lungs were analyzed by immunofluorescence detection of GFP (*green*) and Vimentin or Amphiregulin (*red* and fire scale), white arrowheads indicated metastasis with GFP and Amphiregulin positive staining. Scale bar: 100 µm. Data are expressed as the means ± SD. Mann–Whitney test statistical analysis; * *p* < 0.05.

To investigate the in vivo capability of LXR activation to enhance the invasiveness of PC3 cells, we conducted orthotopic intraprostatic xenografts on NSG mice. As demonstrated in Figure 1D, human prostatic cell lines displayed specific features in terms of EMT profiles when xenografted into NSG mouse prostates. Each batch of mice was treated with DMSO as a vehicle or GW3965 (Figure 7A). After four weeks of treatment, an analysis of the primary implantation site and an assessment of metastatic extension were carried out, representing a preclinical model to monitor invasiveness properties. Chronic treatment with GW3965 did not lead to any notable change in the size of tumors at the implantation site (Figure 7B,C). However, PC3 cells exposed to GW3965 treatment exhibited an architecture and organization typical of mesenchymal cells, characterized by an elongated shape and a greater accumulation of Vimentin compared to those from DMSO-treated mice (Figure 7D). A quantification of the cell circularity confirmed that cells from tumors treated with GW3965 exhibit a spindle phenotype with the higher accumulation of Vimentin (Figure 7E,F). Regarding metastatic dissemination, the number of GFP+ intraperitoneal lymph nodes significantly increased in animals treated with GW3965 (Figure 8A,B), reflecting an enhanced colonization capacity of PC3 cells when LXRs are activated. This observation was confirmed by a substantial increase in pulmonary invasion due to GW3965 treatment (Figure 8C). The analysis of pulmonary metastatic foci revealed a high accumulation of Vimentin (Figure 8D). Consistent with the results obtained in cell culture, GW3965 treatment led to a significant accumulation of Amphiregulin in the lung metastases of animals treated with GW3965 compared to those treated with DMSO (Figure 8E). Altogether, these results demonstrate that GW3965 treatment stimulates the metastatic dissemination of PC3 cells, accompanied by an accumulation of Vimentin and Amphiregulin proteins.

## 4. Discussion

EMT is a process closely linked to cellular metabolism, particularly that of lipids. A good example is the hepatocellular carcinoma in which TGFβ facilitates the use of free fatty acids and therefore the entry of acetyl-CoA into the TCA cycle to provide an energy supply for tumor cell growth [29]. In parallel, related to prostate cancer, cholesterol could control EMT through its binding to the adipocyte plasma membrane-associated protein (APMAP). This binding leads to the inhibition of EGFR internalization, thus causing constitutive activity of ERK1/2 [30]. Considering these reports and the central role of LXRs in lipid homeostasis, exploration of their involvement in EMT regulation is rational. LXRs are commonly associated with anti-tumoral properties in PCa. Previous research demonstrated their roles in suppressing proliferation during the early phases of prostate cancer [18,31,32]. Furthermore, various studies showed the inhibitory effect of LXRs on EMT [14,15]. Considering these observations, we investigated the impact of LXR activation on EMT in advanced prostate cancer models. Our findings challenge the traditional paradigm of LXRs solely serving an anti-EMT role. Instead, our observations suggest that LXRs may assume a rather opposite role, as promoters of EMT during the advanced stages of prostate cancer. These findings correlate with previous results described in gastric cancer cells where LXRs promote invasion and EMT [33], indicating the complex role of those receptors regarding the physio-pathological context. We showed that LXRs enhance Vimentin accumulation in the PC3 cell line without affecting *VIM* gene expression. Additionally, LXR activation increases the expression and accumulation of Amphiregulin. Finally, in an immunocompromised murine model, treatment with an LXR agonist accentuates the aggressive phenotype of PC3 cells, resulting in an increased number of metastases. These findings challenge the conventional understanding of LXRs as purely anti-tumorigenic effectors and emphasize their context-dependent roles, particularly in the advanced stages of prostate cancer, where they may contribute to EMT and metastatic progression. At the molecular level, LXR activation significantly increases Vimentin protein accumulation independently of gene expression modifications. This suggests that the effects of LXR on Vimentin may not result from direct transcriptional regulation but rather from post-translational mechanisms. Several post-translational modifications govern Vimentin stability and function. Phosphorylation in specific serine residues is critical in Vimentin filament disassembly [34,35,36]. The treatment of BHK-21 cells with calyculin-A (cl-A), a phosphatase inhibitor, results in notable Vimentin accumulation and filament disassembly [36]. SUMOylation and ubiquitination also regulate Vimentin protein accumulation [37]. Lastly, the E3 ligase RNF208 targets Vimentin for degradation [38]. Hence, LXR activation could potentially influence one or more of these processes to regulate Vimentin accumulation in PC3 cells. Regarding phosphorylation, interactions between LXR and PKA have been identified [39]. Given that PKA phosphorylates Vimentin, reducing its accumulation, LXRs could potentially act as antagonists to PKA, inhibiting Vimentin phosphorylation and thus leading to increased Vimentin accumulation, thereby stimulating migration, invasion, and metastatic dissemination. Another hypothesis could target Vimentin SUMOylation status, as LXRs are known to influence this process. Li et al. demonstrated that LXR activation delays the degradation of IκBα and NF-κB activity by directly inhibiting SUMOylation [37]. By manipulating this process in PC3 cells, LXRs might regulate Vimentin. Lastly, RNF208 is directly implicated in Vimentin degradation and has been linked to the aggressiveness of various tumor cell lines, with its loss enhancing dissemination and metastasis spreading [38]. LXRs may act as negative regulators of RNF208, reducing Vimentin protein degradation and thereby explaining the observed increase in Vimentin protein without a corresponding rise in mRNA transcripts. This work highlights a new putative target of LXR. We demonstrated that LXR activation in PC3 cells increases both AREG expression and the corresponding Amphiregulin protein accumulation. Amphiregulin belongs to the epidermal growth factor (EGF) family. It is constitutively expressed in various epithelial and mesenchymal cells and is involved in tissue development and homeostasis [27]. It serves as the primary autocrine growth factor for keratinocytes, the cells comprising the epidermis [40]. Amphiregulin emerges as a significant player in the EMT process. In cancer, extensive research on Amphiregulin has emerged since the 2010s. AREG is overexpressed in various tumor types and is a poor prognostic marker [16,41,42,43,44,45,46]. Serum Amphiregulin levels are associated with reduced survival in lung cancer patients [16]. Amphiregulin can induce EMT in a tumor context. The suppression of AREG has been observed to inhibit the migration and invasion of AsPC-1 cells, a pancreatic cancer cell line. Furthermore, its loss leads to increased E-cadherin expression and the inhibition of Vimentin, Snail, and Slug levels. In PANC-1 cells, another pancreatic tumor cell line, AREG stimulation increased cell migration, invasion, and the expression of EMT transcription factors [28]. In prostate cancer, serum Amphiregulin concentrations significantly increase after androgen deprivation [47]. Its overexpression in LNCaP, DU145, and PC3 cell lines is associated with a substantial gain in migration and invasion [48]. Collectively, these data suggest that Amphiregulin likely plays a role in PC3 cells’ aggressive phenotype acquisition through LXR activation. A pending hypothesis remains that the putative regulation of Vimentin accumulation in response to LXR activation could be assumed by Amphiregulin. To test this possibility, we performed AREG knockdown in PC3 cells to monitor Vimentin accumulation in response to GW3965 treatment (Figure S2). We observed that GW3965 induction leads to increased Vimentin accumulation even in the absence of AREG, thus indicating that the regulation of Vimentin and Amphiregulin by LXRs in PC3 cells is the result of independent mechanisms. 

## 5. Conclusions

This work demonstrates the involvement of LXRs in advanced PCa, modulating EMT. Therefore, the pharmacological targeting of LXRs in various stages of the disease needs to be carefully addressed given their activation leads to the opposite effect regarding cancer history.

## Figures and Tables

**Table 1:** Antibody list and references.

Antigen	Source	WB Working Dilution	IF Working Dilution	Reference	Manufacturer
Fibronectin	Rabbit	1/1000	1/200	Ab2413	Abcam Cambridge UK
Vimentin	Rabbit	1/1000	1/200	D21H3	Cell Signaling Technology, Ozyme, Saint-Cyr- l’Ecole, France
N-Cadherin	Rabbit	1/1000	1/200	D4R1H	Cell Signaling Technology
E-Cadherin	Rabbit	1/1000	1/200	610182	BD Transduction Laboratories, Le Pont de Claix, France
Amphiregulin	Rabbit	1/1000	1/200	16036-1-AP	Proteintech, Stockport, UK
GAPDH	Mouse	1/10,000	-	D4C6R	Cell Signaling Technology
GFP	Rabbit	-	1/1000	A11122	Invitrogen, Villebon Sur Yvette, France
Actin	Rabbit	1/10,000	-	A2066	Sigma-Aldrich, Saint-Quentin-Fallavier, France

**Table 2:** List of primers used for RTqPCR analysis.

Gene	Primer Sequence Forward	Primer Sequence Reverse
*CDH2*	TGCCCTCAATGGGATCTTGA	AGGCCATATGTGGGATTGCC
*VIM*	AGAGGAAGCCGAAAACACCC	TTGCAAAGATTCCACTTTGCGT
*FN*	TCCCTCGGAACATCAGAAAC	CAGTGGGAGACCTCGAGAAG
*SNAI1*	CTCTAGGCCCTGGCTGCTAC	GCTTGTGGAGCAGGGACATT
*SNAI2*	TTGTGTTTGCAAGATCTGCGG	GCAAATGCTCTGTTGCAGTGA
*ZEB1*	ATCCTGGGGCCTGAAGCT	TGGTGTGCCCTGCCTCTGGT
*CDH1*	ATGAGTGTCCCCCGGTATCT	ACGAGCAGAGAATCATAAGGGGCG
*DSP*	GATCACCGACCAGAACTCGG	AAGTTCTGCACCTGACGCTT
*ABCG1*	CAGGAAGATTAGACACTGTG	GAAAGGGGAATGGAGAGAAG
*SREBF1*	GGAGGGGTAGGGCCAACGGCCT	CATGTCTTCGAAAGTGCAATCC
*NR1H3*	AGGAGTGTCGGCTTCGCAAA	CTCTTCTTGCCGCTTCAGTTT
*NR1H2*	AACAAACGCTCCTTCTCCGA	GGTGATACACTCTGTCTCGT
*AREG*	TCCCCTGTGAGTGAAATGCC	GTTACTGCTTCCAGGTGCTCT

**Table 3:** List of 28 genes commonly deregulated between WTvsDKO mouse prostates and PC3 DMSOvsGW3965 treatment datasets.

ENS	Gene Name	Base Mean	log2FoldChange	FC	lfcSE	Stat	*p*-Value	Padj
ENSG00000120708	TGFBI	0.55747659	1.78535814	3.44704023	1.90412944	0.93762436	0.34843749	NA
ENSG00000109321	AREG	490.424353	1.16715752	2.24568803	0.10414778	11.2067441	3.78 × 10^−29^	9.96 × 10^−26^
ENSG00000140092	FBLN5	13.0413403	0.39755778	1.31727611	0.38800452	1.02462151	0.30554181	NA
ENSG00000115935	WIPF1	14.8540542	0.3936751	1.31373574	0.36984887	1.06442154	0.28713778	NA
ENSG00000115738	ID2	633.907513	0.29972602	1.23091063	0.14101883	2.12543265	0.03355053	0.61122854
ENSG00000166923	GREM1	13.0693234	0.29423341	1.22623323	0.40445516	0.72748092	0.4669314	NA
ENSG00000135919	SERPINE2	2599.06039	0.28987312	1.22253275	0.08467547	3.42334244	0.00061856	0.05965824
ENSG00000167460	TPM4	4732.16738	0.27491589	1.20992355	0.07462908	3.6837634	0.00022982	0.02818358
ENSG00000117152	RGS4	90.6207624	0.259963	1.19744799	0.18138207	1.43323428	0.1517909	0.90553331
ENSG00000164949	GEM	331.016875	0.25879656	1.19648023	0.1070989	2.4164259	0.01567372	0.45082373
ENSG00000085063	CD59	18866.2581	0.25583504	1.19402665	0.06778507	3.77420929	0.00016052	0.02069425
ENSG00000140937	CDH11	7637.94552	0.25487472	1.19323212	0.10960447	2.32540447	0.02005034	0.48799449
ENSG00000122786	CALD1	1470.33888	0.21993651	1.16468233	0.12820702	1.71547945	0.08625736	0.80643802
ENSG00000104332	SFRP1	25.8588417	0.21976976	1.16454772	0.26265947	0.83670983	0.40275569	NA
ENSG00000176788	BASP1	2800.41858	0.21759661	1.16279487	0.11227704	1.93803304	0.05261919	0.70623589
ENSG00000170558	CDH2	4236.84196	0.19929013	1.14813328	0.1124252	1.77264635	0.07628733	0.7912538
ENSG00000065534	MYLK	1140.93967	0.19162924	1.14205271	0.09693455	1.9768931	0.04805372	0.68797265
ENSG00000026025	VIM	12641.7501	0.16067385	1.11780912	0.08217763	1.95520179	0.05055926	0.70112503
ENSG00000166741	NNMT	1964.88143	0.14971687	1.10935174	0.10141317	1.47630602	0.13986176	0.8928307
ENSG00000073712	FERMT2	4345.12245	0.14738122	1.10755721	0.05953327	2.47561126	0.01330083	0.41427885
ENSG00000145147	SLIT2	2457.46308	0.12358147	1.08943602	0.11657471	1.06010534	0.28909668	0.96733795
ENSG00000128595	CALU	7375.79195	0.11542364	1.0832931	0.07137564	1.61712924	0.10585039	0.85045867
ENSG00000112715	VEGFA	1458.65656	0.10757865	1.07741843	0.0978457	1.0994725	0.27156202	0.96186384
ENSG00000163069	SGCB	2110.832	0.09116566	1.06523051	0.07435268	1.22612481	0.22015171	0.94409558
ENSG00000113140	SPARC	7116.58414	0.06749718	1.04789719	0.3734534	0.1807379	0.85657331	0.99872891
ENSG00000164176	EDIL3	3250.12928	0.04869492	1.03432883	0.10049241	0.48456319	0.62798623	0.99872891
ENSG00000111799	COL12A1	5108.27642	0.04519455	1.03182231	0.08228866	0.54921966	0.58285471	0.99872891
ENSG00000106483	SFRP4	3.74887888	−0.1991432	0.8710677	0.79958454	−0.2490584	0.80331561	NA

## Data Availability

GEO accession GSE245578.

## Supplementary Materials

The following supporting information can be downloaded at: https://www.mdpi.com/article/10.3390/cancers16162776/s1, Figure S1: Non-cropped prostate section of NSG-grafted with LNCaP, DU145 or PC3 cells; Figure S2: Vimentin accumulation in response to GW3965 is not affected by Amphiregulin knockdown in PC3 cells; File S3: Original western blots.
