# Supplementary material for: Liver X Receptors Enhance Epithelial to Mesenchymal Transition in Metastatic Prostate Cancer Cells
Source: Cancers (Basel). 2024 Aug 6;16(16):2776. doi: 10.3390/cancers16162776 (PMC11353074; doi:10.3390/cancers16162776)
Supplement: Supplementary file 1 [file cancers-16-02776-s001.zip › cancers-3075539-File S1.pdf]

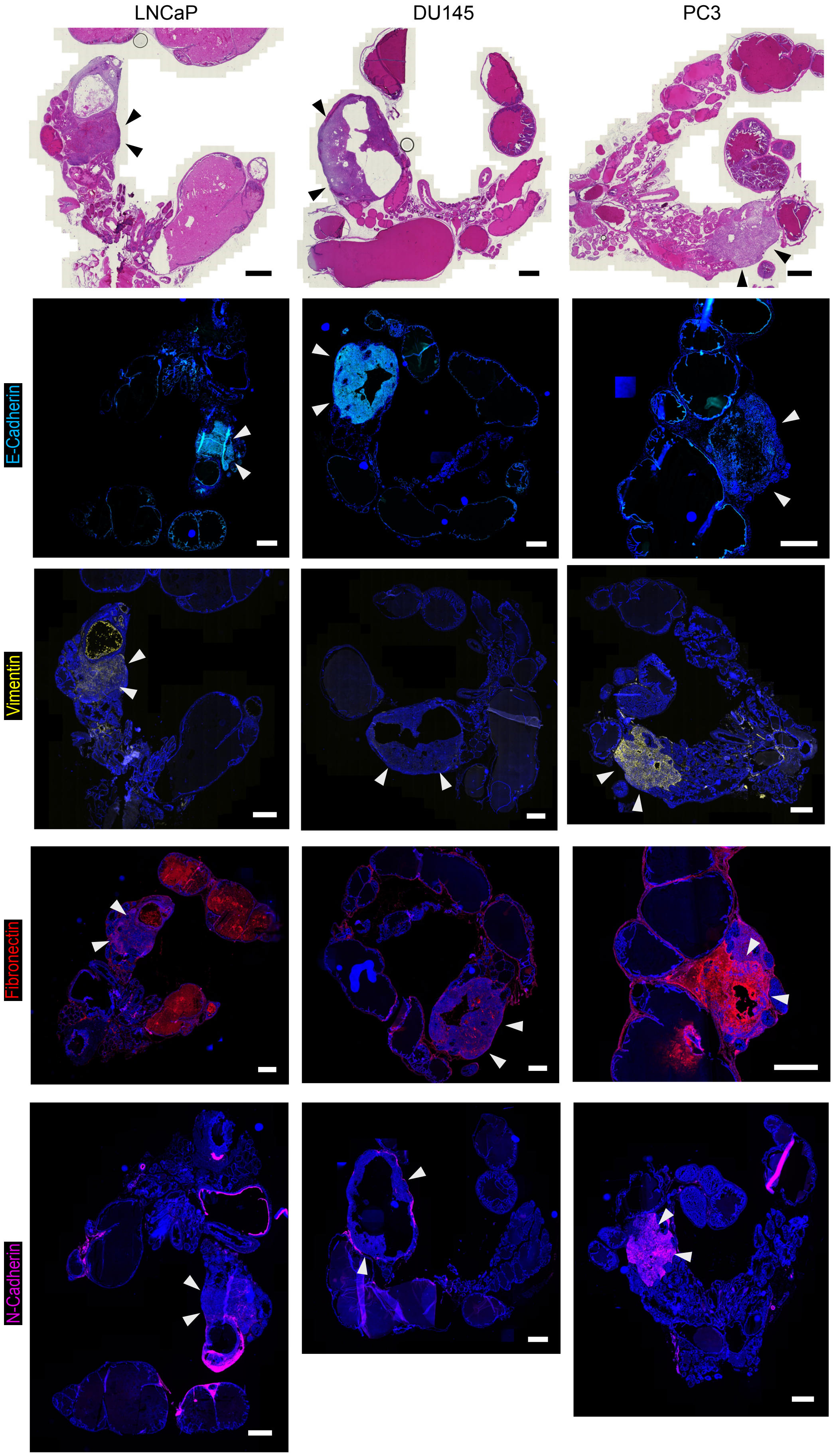

Figure S1: Non-cropped prostate section of NSG-grafted with LNCaP, DU145 or PC3 cells.
Immunofluorescence of LNCaP, DU145 and PC3 cells stained for E-cadherin (*blue*), Fibronectin (*red*), Vimentin (*yellow*) and N-Cadherin (*purple*). Scale bar: 100 µm.
