# Supplementary material for: Liver X Receptors Enhance Epithelial to Mesenchymal Transition in Metastatic Prostate Cancer Cells
Source: Cancers (Basel). 2024 Aug 6;16(16):2776. doi: 10.3390/cancers16162776 (PMC11353074; doi:10.3390/cancers16162776)
Supplement: Supplementary file 1 [file cancers-16-02776-s001.zip › cancers-3075539-File S2.pdf]

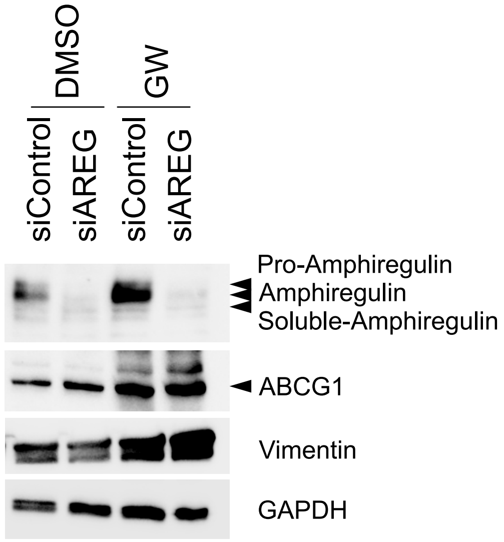

Figure S2: Vimentin accumulation in response to GW3965 is not affected by Amphiregulin knock down in PC3 cells. Western blots of Amphiregulin, ABCG1 and Vimentinaccumulation in PC3 cells treated with DMSO or GW3965 for 48 hrs. Amphiregulin silencing has been performed 24 hrs prior DMSO and GW3965 induction. GAPDH accumulation was used as a loading control.
