# Supplementary figures and images for: Liver X Receptors Enhance Epithelial to Mesenchymal Transition in Metastatic Prostate Cancer Cells
Source: Cancers (Basel). 2024 Aug 6;16(16):2776. doi: 10.3390/cancers16162776 (PMC11353074; doi:10.3390/cancers16162776)

FIGURE 1

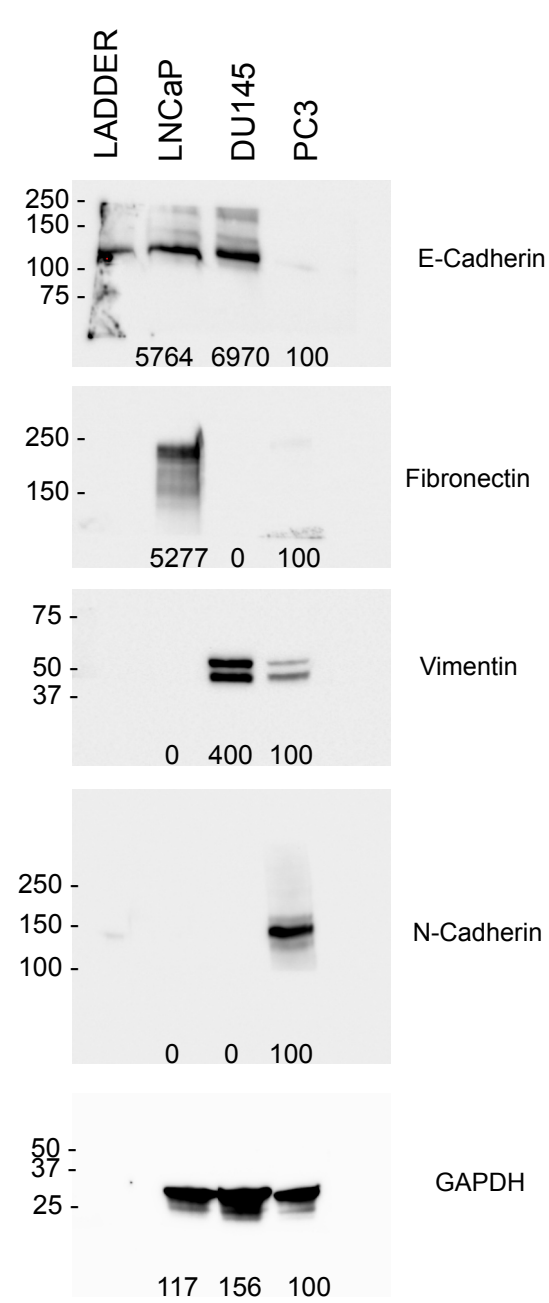

FIGURE 5

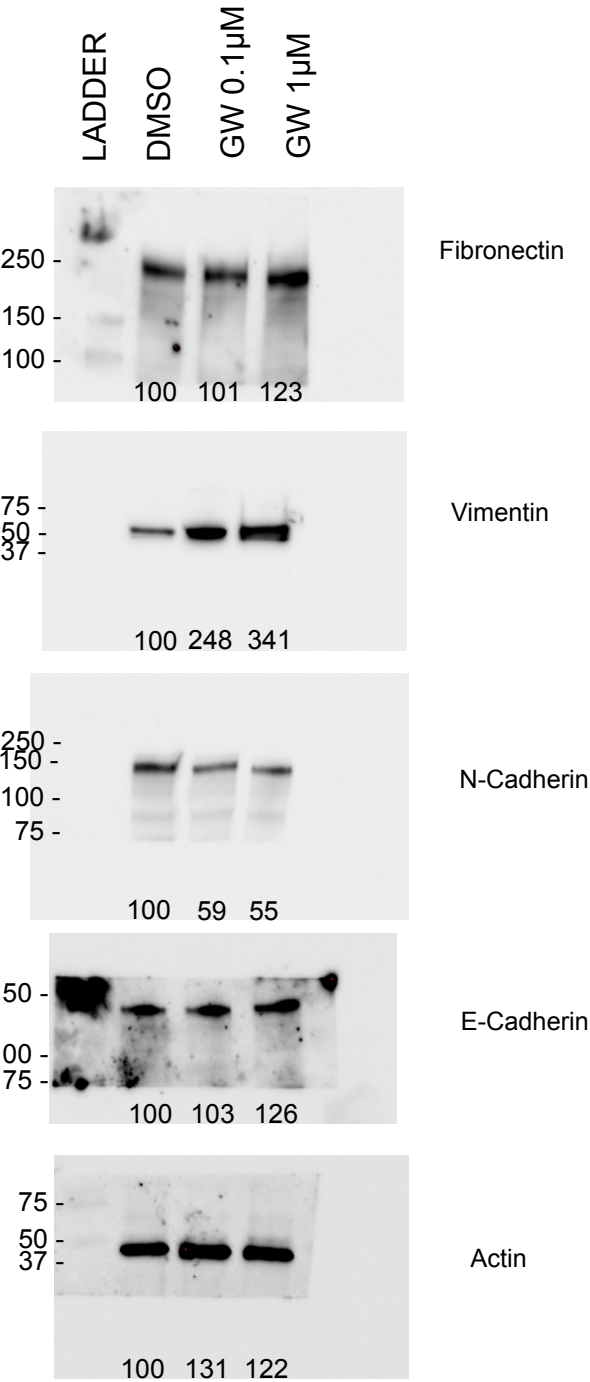

FIGURE 6

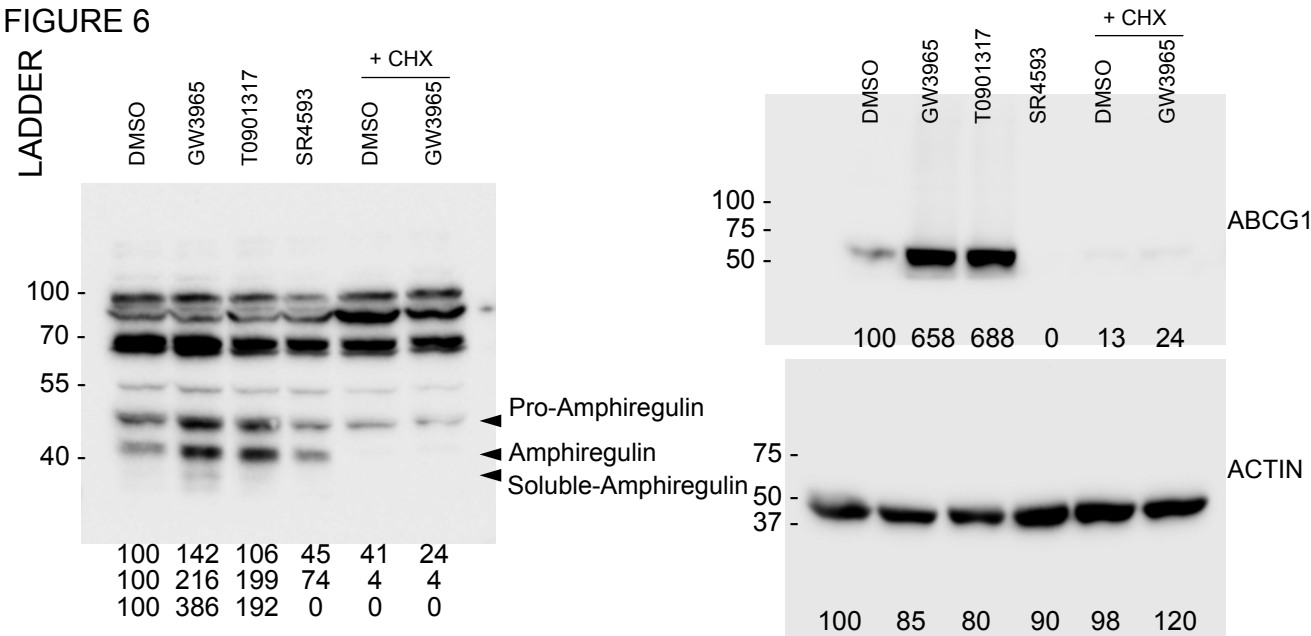

Supplement: Supplementary file 1 [file cancers-16-02776-s001.zip › cancers-3075539-File S3.pdf]
